# Arthroscopic Tenodesis Through Positioning Portals to Treat Proximal Lesions of the Biceps Tendon

**DOI:** 10.1007/s12013-014-0071-9

**Published:** 2014-09-20

**Authors:** Ji Shen, Qing-feng Gao, Yao Zhang, Yao-hua He

**Affiliations:** 1 Department of Orthopedics, Shanghai Jiao Tong University Affiliated Sixth People’s Hospital, Shanghai, 200233 P.R. China; 2 Department of Orthopedics, Shanghai Sixth People’s Hospital, Soochow University, Shanghai, P.R. China

**Keywords:** Biceps tenodesis, Portal, Suture anchors, Landmark, Arthroscopy

## Abstract

**Abstract:**

Arthroscopic biceps tenodesis is a good choice for treating proximal lesions of the biceps tendon. However, there are few descriptions of the surgical approach. We introduce a technique for proximal biceps tenodesis using positioning portals and placing suture anchors. Our patients had a minimum of 12 months of follow-up. Between January 2010 and June 2012, a total of 49 patients (21 men, 28 women) underwent arthroscopic biceps tenodesis. The pathology was mainly associated with proximal lesions of the biceps tendon, with the diagnosis confirmed in all patients. Patients were evaluated preoperatively and then up to and including the final follow-up. Their pain and conditions were assessed using the Constant, American Shoulder and Elbow Surgeons (ASES), and University of California at Los Angeles (UCLA) scores for pain; range of active forward flexion; and active range of motion. All data were analyzed statistically. All patients were operated on successfully. They achieved good healing during the follow-up (mean 14 months; range 12–34 months). Before surgery the ASES, Constant, and UCLA scores were 17.0, 39.4, and 15.4, respectively. After surgery they were 33.6, 89.1, and 31.2, respectively. The scores had significantly improved: ASES scores from 17.0 to 33.6 (*P* < 0.05); Constant scores from 39.4 to 89.1 (*P* < 0.05); UCLA scores from 15.4 to 31.2 (*P* < 0.05). Arthroscopic tenodesis through positioning portals to treat proximal lesions of the biceps tendon produces satisfactory clinical outcomes. This technique is convenient and safe.

**Level of evidence:**

Level IV, Case Series, Treatment Study.

## Introduction

Disorders of the proximal biceps tendon are common causes of shoulder pain and dysfunction [1]. Many authors believe that the long head of the biceps brachii tendon is a major contributor to shoulder pain [2–4] because it spans an intra-articular portion and an extra-articular segment, leading to multiple possibilities for pathology of the tendon itself [5]. Patients who develop disorders of the proximal biceps experience persistent anterior shoulder pain and have flexion or extension deficit. These disorders seriously affect the quality of life. Treatment of disorders of the proximal biceps, therefore, should not be delayed. Because traditional surgical treatment does not address the intra-articular portion of the tendon [6], arthroscopic biceps tenodesis has been proposed for treating disorders of the proximal biceps.

Previous reports have not described where to place the portals. When the biceps tenodesis is performed through a completely arthroscopic technique, however, portal selection is one of the most important factors that affect the success or failure of the operation. The portals should provide safe, convenient placement sites for suture anchors.

Because we were familiar with the technique of arthroscopic repair of rotator cuff tears using suture anchors, we were able to develop a new way to make portals for proximal biceps tenodesis. The standard arthroscopic portals—anterior, posterior, anterolateral, lateral, and Neviaser—do not allow proximal biceps tenodesis. We, therefore, introduced the most appropriate portals required for proximal biceps tenodesis using positioning portals to apply suture anchors.

## Materials and Methods

### Patients

**Fig. 1:**
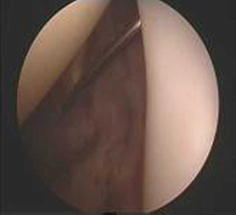
Spinal needle is placed in the biceps tendon in the glenohumeral joint

**Fig. 2:**
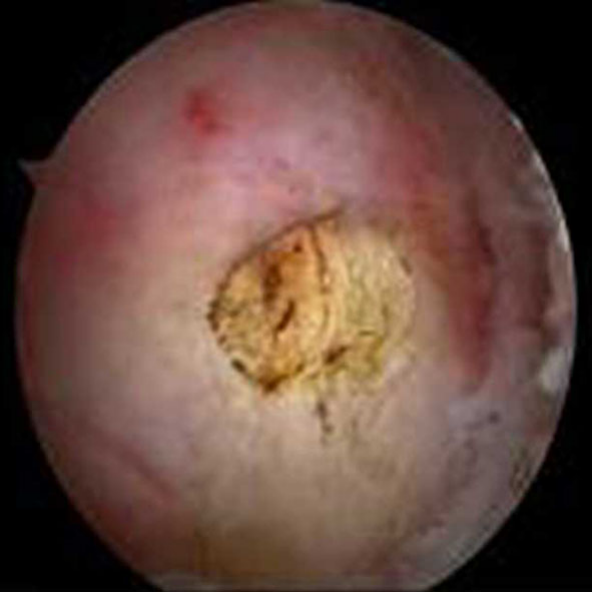
Spinal needle was found under the subacromial space

Between January 2010 and June 2012, a total of 49 patients (21 men, 28 women) underwent arthroscopic biceps tenodesis through portals specifically positioned to treat proximal lesions of the biceps tendon. All patients were followed up at a mean of 14 months (range 12–34 months). Patients were selected after undergoing preoperative physical examination and magnetic resonance imaging (MRI). The indication was confirmed intraoperatively during arthroscopy (Figs. 1, 2). The patients had different levels of subacromial impingement, but no rotator cuff tears. In each case, it was confirmed that a proximal biceps tendon disorder was the main factor causing symptoms. All patients had previously failed appropriate nonoperative therapy.

The disorders of proximal biceps tendon included tendinitis (*n* = 39), rupture (*n* = 5), subluxation (*n* = 2), and a superior labral tear from anterior to posterior (SLAP lesion) (*n* = 3). The mean age of the patients was 56 years (range 37–65 years). All patients were evaluated preoperatively and during follow-up to the final follow-up using the Constant and Murley [7], American Shoulder and Elbow Surgeons (ASES) [8], and University of California at Los Angeles (UCLA) [9] scores for pain; range of active forward flexion; active range of motion, and strength of the biceps. The data were analyzed statistically. Postoperative MRI scans were obtained for most of the patients.

### Surgical Technique

The patient was placed in a lateral decubitus position. The arm was suspended at approximately a 45° angle of abduction and 15° forward flexion. The traction on the shoulder joint was ~4 kg. A pressure apparatus was routinely used with two bags of physiological saline. The pressure was ~40 mmHg.

**Fig. 3:**
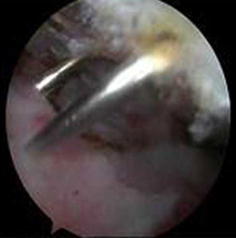
Biceps tendon is directly under the mark

The anterior, posterior, anterolateral, and lateral portals were placed to obtain visualization of anatomical structures and defects. The arthroscopy sheath and blunt obturator were inserted into the glenohumeral joint through the posterior portal. The glenohumeral joint was then explored with the 30° arthroscopic to identify any disorders of the proximal biceps tendon such as tendinitis, rupture, subluxation, or instability. When other structures were confirmed to be normal, a needle test was conducted in the area of the bicipital groove. Once the spinal needle was inserted into the biceps tendon, we were able to see it through the arthroscope in the posterior portal (Fig. 3). The location of the spinal needle was usually just medial to the lateral part of the greater tuberosity. The arthroscope, still in the posterior portal, was then removed from the joint and reoriented under the acromion into the subacromial bursa. Using a motorized shaver through the lateral portal and the anterior portal, subacromial decompression was performed, which relieved shoulder impingement.

**Fig. 4:**
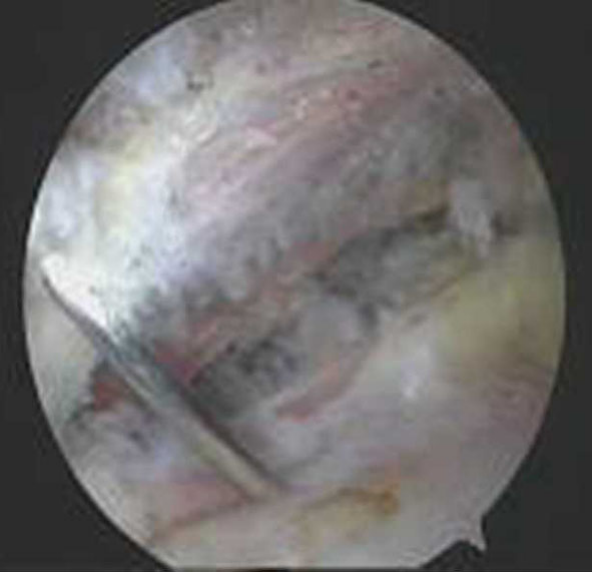
Second spinal needle was inserted proximal to the bicipital groove according to the first one

The arthroscope was then removed from the posterior portal and placed in the lateral portal, and subacromial decompression was continued. Acromioplasty is a simple and effective technique for preventing subacromial impingement syndrome and should be done first. The arthroscope was still in the lateral portal. Using the motorized shaver, we then cleared the anterior part of the bursa and inflammatory tissues through the anterior portal. All adventitial tissues were removed until we achieved an excellent view of the rotator cuff tendon and the subacromial space. A clear field of vision during the procedure prevents abutment of the instrumentation, while the spinal needle was being used (Fig. 4).

**Fig. 5:**
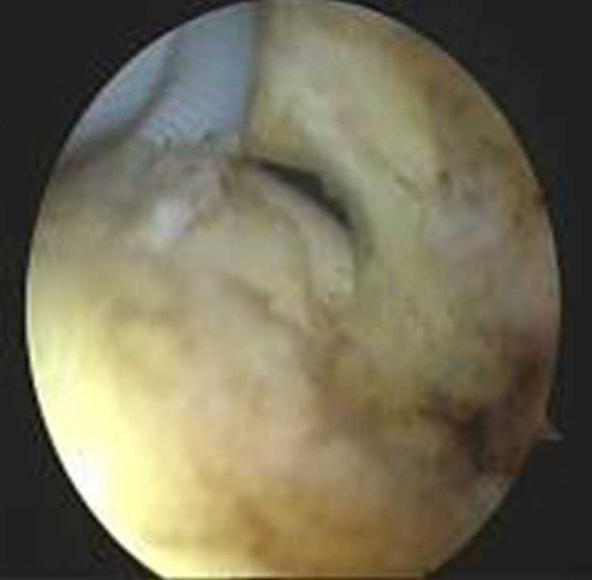
Biceps tendon was exposed

**Fig. 6:**
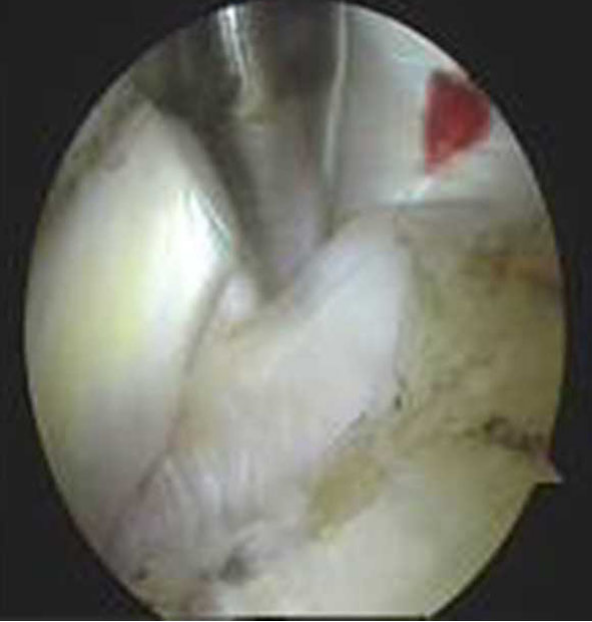
Probe was used to move the biceps tendon to show the bicipital groove

Keeping the arthroscope in the lateral portal, the anterior portal became the working portal. Using an electrocautery ablation device, we made a mark at the site where we would place the spinal needle. The mark was made to locate the bicipital groove (Fig. 5). The biceps tendon was directly under the mark. A second spinal needle was used to perform a needle test lateral to the first needle and 2 cm distal to it. The second spinal needle was inserted proximal to the bicipital groove (Fig. 6). The needles should be perpendicular to the bicipital groove. Once the needles had been placed in the best positions, they became perfect landmarks for creating other portals. At this point, two 3-mm incisions were made based on the landmarks. Use of the portals to establish intraoperative locations was completed.

**Fig. 7:**
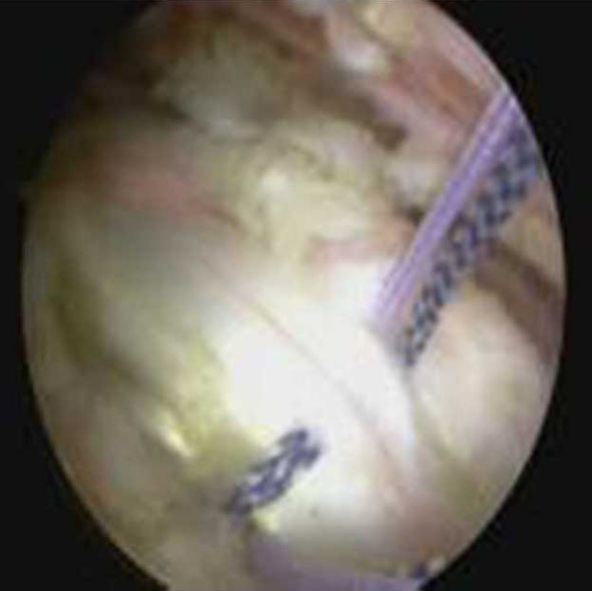
One of the sutures is placed through the biceps tendon and the other is not

**Fig. 8:**
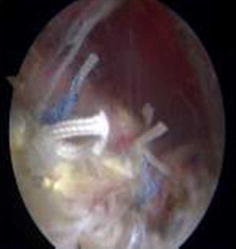
Strong fixation is achieved in the biceps tendon

All portals were now either a working portal or a viewing portal. The portal made from the first spinal needle was the main working portal. The arthroscopic equipment was then transferred to the portals for the intraoperative locations. The mark labeling the bicipital groove was easily found through the viewing portal. An electrocautery ablation device was then used to clean the tissue around the mark. Care was taken to avoid injuring the rotator cuff tendons. When the transverse humeral ligament tissue had been removed, the biceps tendon was exposed (Fig. 7). After sufficient exposure was attained, a probe was used to move the bicep tendon to show the biceps groove (Fig. 8). Then, at the middle of the biceps groove, the bony surface under the biceps tendon was abraded with a bur. Two suture anchors were passed into the new working portal and embedded in the fresh bone bed. The position of the fresh bone bed was proximal to the narrowest point of the bicipital groove. Two suture anchors were placed 2 cm apart.

**Fig. 9:**
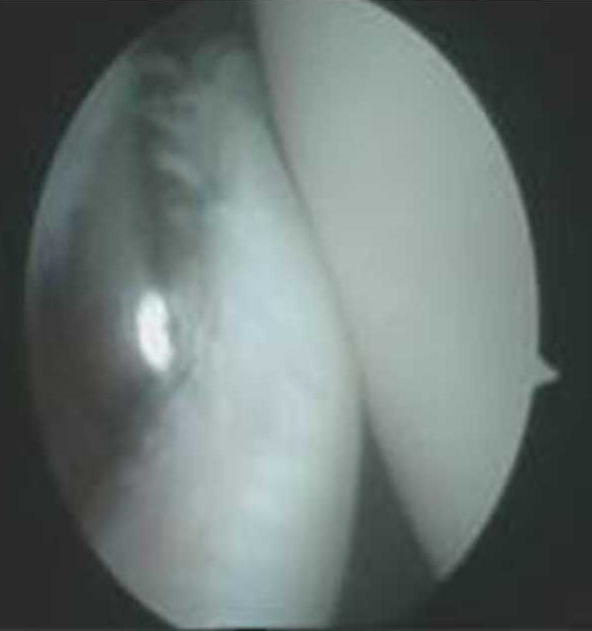
Shaver was used to remove the remaining tendon

**Fig. 10:**
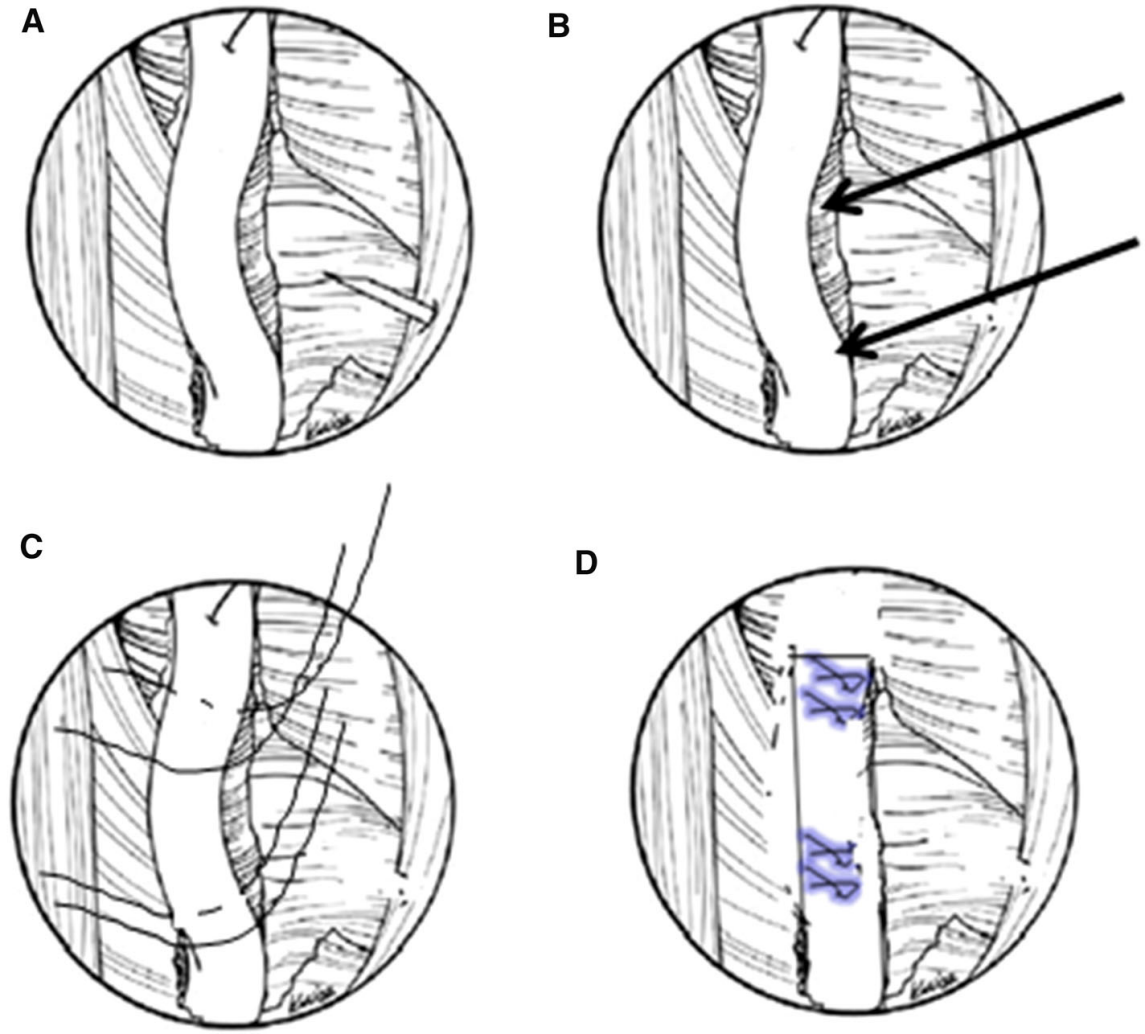
Concept of the surgical technique. **a** Biceps tendon is exposed. **b** Suture anchors (*arrows*). Two suture anchors are 2 cm apart. **c** One of the sutures is placed through the biceps tendon and the other is not. **d** Strong fixation is achieved in the biceps tendon

**Fig. 11:**
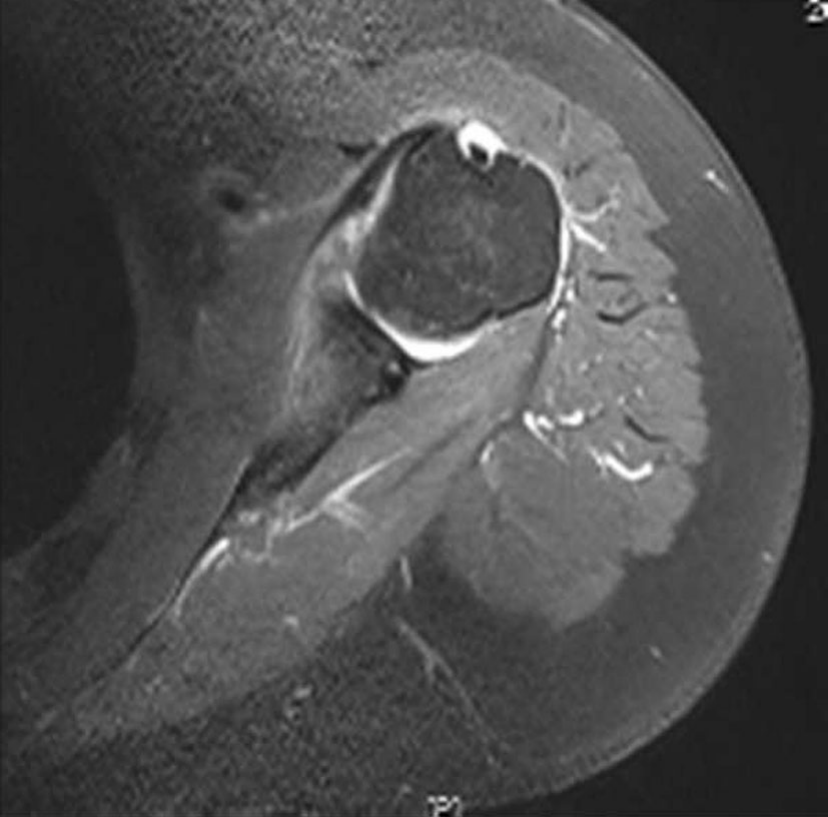
T2-weighted magnetic resonance (MRI) axial view of a diseased biceps tendon

**Fig. 12:**
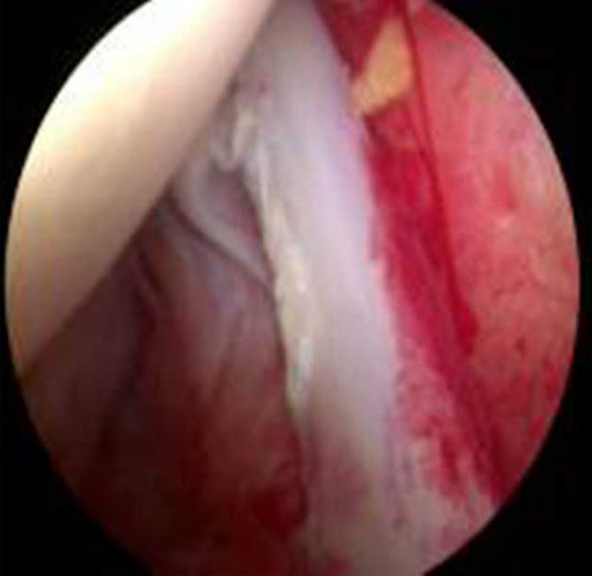
Arthroscopic view of tendinitis causing significant discomfort to the patient

Each suture anchor has two sutures: one is placed through the biceps tendon, and the other is not (Fig. 9). All sutures were then pulled out from their particular portal with a crochet hook. Placement of another suture anchor completed the procedure. The sutures were each tied with a Tennessee slider and four alternating half-hitches. The knots and loops were checked to make certain they were secure and that the biceps tendon had strong fixation (Fig. 10). The residual intra-articular biceps tendon was released near the sutures using an electrocautery ablation device. The remaining tendon was excised at the superior glenoid labrum using a basket punch and removed using a shaver (Fig. 11). The operation was now finished. The concept of the surgical technical is outlined in Fig. 12.

### Rehabilitation

All patients wore a sling from the day of surgery until 6 weeks postoperatively. Passive pendulum exercises were allowed during the first 2 weeks. After 2 weeks, passive motion was allowed for the shoulder and wrist without restriction or immobilization. Active elbow flexion was prohibited for about 6 weeks to allow healing of the tenodesis. Full passive range of motion was undertaken after 6 weeks along with removal of the sling. After 6–8 weeks, active elbow flexion exercises were slowly incorporated into the rehabilitation program, with strengthening delayed. After 3 months, gradual strengthening was performed until good long-term function was established.

## Results

All patients were operated on successfully, with good healing. They were followed up at a mean of 14 months (range 6–34 months). Before surgery, the ASES, Constant, and UCLA scores were 17.0, 39.4, and 15.4, respectively. After surgery, the scores were 33.6, 89.1, and 31.2, respectively. These outcomes demonstrate that all scores had significantly improved: ASES scores from 17.0 to 33.6 (*P* < 0.05); Constant scores from 39.4 to 89.1 (*P* < 0.05); and UCLA scores from 15.4 to 31.2 (*P* < 0.05) (Table 1).

**Table 1:** UCLA score ($\overline{\text{x}}$ ± *s*,*n* = 21) and Constant score ($\overline{\text{x}}$ ± *s*, *n* = 21)

UCLA score ($\overline{\text{x}}$ ± *s*,*n* = 21)					
Time	Pain	Function score	Active forward flexion	Anteflexion strength	Total
Preoperative	1.1 ± 0.3	3.4 ± 1.8	2.0 ± 0.2	2.0 ± 0.8	8.7 ± 2.5
The final follow-up time	8.3 ± 1.5	7.0 ± 2.7	4.9 ± 0.4	4.3 ± 0.6	30.4 ± 4.5
*T* value	−21.916	−6.67	−30.50	−10.954	−22.186
*P* value	0.000	0.000	0.000	0.000	0.000

Constant score ($\overline{\text{x}}$ ± *s*, *n* = 21)								
Time	Pain	Activities of daily living	Arms forward	Forward elevation	Abduction strength	Active external rotation	Internal rotation	Abduction
Preoperative	3.6 ± 3.5	2.8 ± 1.8	5.0 ± 1.3	4.3 ± 1.2	9.6 ± 4.0	4.38 ± 1.6	3.6 ± 1.6	3.9 ± 1.3
The final follow-up time	11.2 ± 2.2	9.2 ± 1.6	9.5 ± 1.0	9.3 ± 1.0	19.4 ± 4.5	9.3 ± 0.9	9.5 ± 1.8	9.6 ± 0.8
*T* value	−6.776	−13.564	−13.364	−15.138	−8.516	−10.527	−16.812	−18.0
*P* value	0.000	0.000	0.000	0.000	0.000	0.000	0.000	0.000

**Fig. 13:**
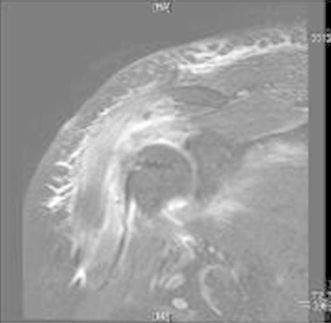
MRI appearances of the suture anchor and final tenodesis

**Fig. 14:**
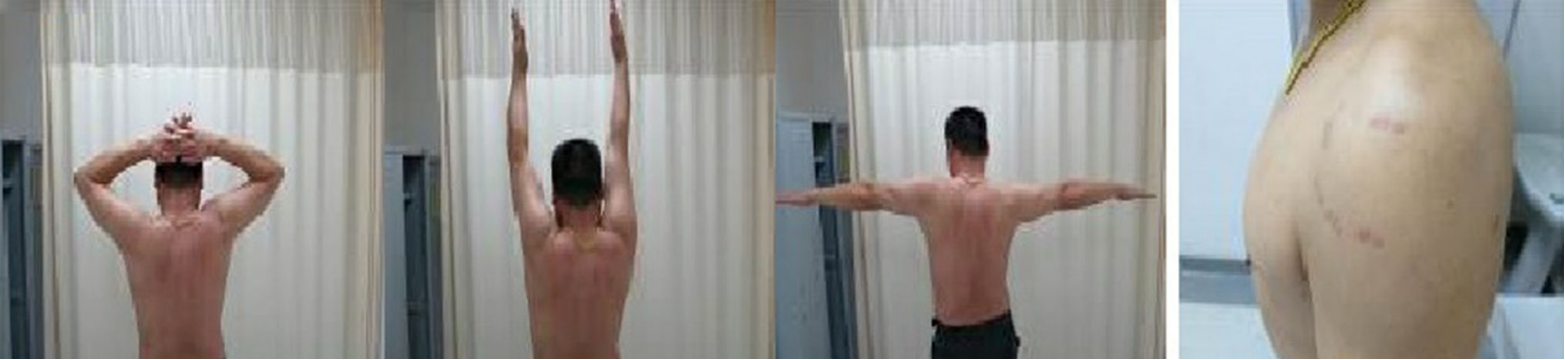
Photographs show good functional recovery in a 42-year-old man 1 year after surgery

The contour of the biceps after surgery was not different from that preoperatively in any of the patients. MRI evaluation showed good healing of the biceps tendon in the groove (Fig. 13). There were no serious complications. Elbow movement and biceps strength were slightly decreased, but it did not affect the patients’ normal life activities. One male patient has been in constant pain since the operation for unexplained reasons. Otherwise, 98 % of the patients regained good shoulder function, and 100 % were satisfied with the cosmetic results (Fig. 14).

## Discussion

The isolated biceps pathology mainly includes tendinitis, rupture, subluxation or instability, pulley lesions, and SLAP lesions [10]. The probability of isolated biceps pathology is about 5 %. It is often associated with subacromial impingement [11]. Thus, the diagnosis and treatment of proximal biceps tendon injuries continue to be a challenge. Arthroscopic biceps tenodesis provides many advantages. The operation is less invasive, it provides better cosmesis, and there is no loss of elbow movement or biceps strength. Arthroscopic biceps tenodesis established a new lengthwise biceps attachment, so the length–tension relation of the biceps muscle can be maintained. Tenodesis represents an especially reasonable alternative for the younger, relatively active patient.

Gartsman first reported the technique of arthroscopic biceps tenodesis in 2000. Since then, many authors have described the procedure. The fixation techniques—interference screw fixation, suture anchor fixation, and so on—have allowed patients to acquire satisfying therapeutic effects and excellent functional recovery [3, 12–17].

We believe that arthroscopic biceps tenodesis is a good procedure for treating proximal lesions of the biceps tendon, although there are few reports on this surgical approach. One of the recent controversies addressed the problem of how to place the most appropriate portals for arthroscopic biceps tenodesis [18, 19]. Appropriate portals are important if biceps tenodesis is to be performed easily using the arthroscopic technique. The senior author described the most appropriate portals for arthroscopic biceps tenodesis and reported the preliminary clinical results.

**Fig. 15:**
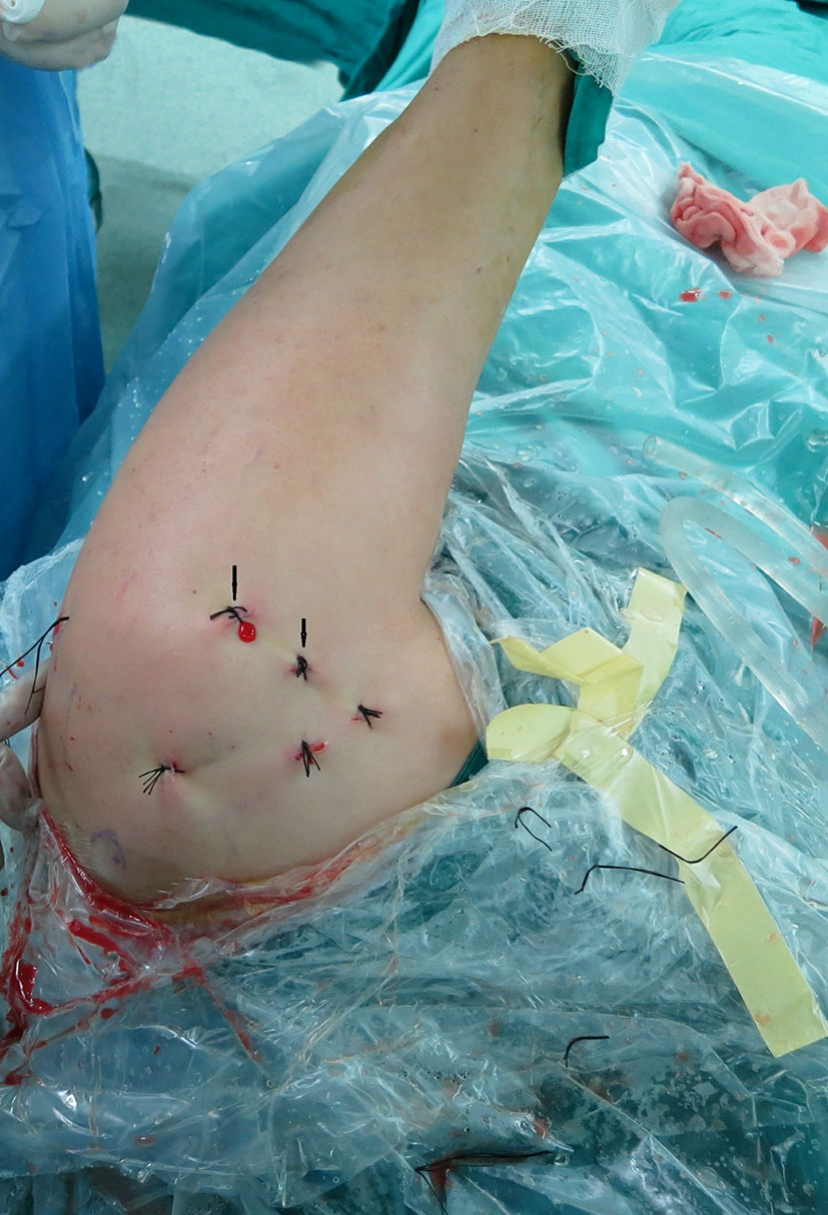
Positioning portals (*arrows*)

When arthroscopic tenodesis is performed through portals positioned properly for treating proximal lesions of the biceps tendon, it has several advantages beyond the distinct advantages of tenodesis itself. First, suture anchors have often been used during arthroscopic repair of rotator tears, so we know that fixation with suture anchors provides strong initial fixation. Because one of the sutures is through the biceps tendon and the other is not, biceps tendon avulsions cannot occur. Second, a needle test can be used to create portals quickly and exactly. The positioning portals provide an optimal angle for suture anchor placement near the greater tuberosity. The suture anchors can, therefore, be directly embedded in an ideal place. Third, the portals were made based on the anatomy zone of the biceps groove (Fig. 15), ensuring that the portals can be used safely and are sufficiently far away from vital structures such as axillary nerves and important arteries and veins. Fourth, it is easy to provide the patient with a safe operation and early mobilization. Fifth, the procedure requires only five or six small portals, so patients experience early recovery and minimal scars.

## Conclusions

The authors do not state that arthroscopic biceps tenodesis is superior to simple tenotomy—it is simply a new biceps tenodesis technique. Being able to identify the correct surgical exposure portals have often led to improved outcomes and possibly decreased surgical time. The use of a position portals device to provide an optimal angle for suture placement along the articular edge near the greater tuberosity takes advantage of an already existing technology. The use of position portals may accomplish several improvements over current accepted treatment. The technology is less difficult than other techniques. Position portals allow us to find the tendon quickly, which saves time and avoids frustration. Arthroscopic biceps tenodesis requires advanced and complicated arthroscopic skills. We described the technique fully to help make it more attractive.
